# Finding the event structure of neuronal spike trains

**DOI:** 10.1186/1471-2202-12-S1-P333

**Published:** 2011-07-18

**Authors:** Vincent J Toups, Jean-Marc Fellous, Peter J Thomas, Terrence J Sejnowski, Paul H Tiesinga

**Affiliations:** 1 Department of Physics & Astronomy, University of North Carolina, Chapel Hill, NC 27599, USA; 2 Psychology Department, University of Arizona, Tucson, AZ 85721, USA; 3 Departments of Mathematics, Biology and Cognitive Science, Case Western Reserve University, Cleveland, OH 44106, USA; 4 Department of Neuroscience, Oberlin College, Oberlin, OH 44074, USA; 5 Howard Hughes Medical Institute, The Salk Institute, La Jolla, CA 92037, USA; 6 Division of Biological Sciences, University of California San Diego, La Jolla, CA 92037, USA; 7 Donders Institute for Brain, Cognition and Behaviour, Radboud University Nijmegen, Nijmegen, 6525 AJ, The Netherlands

## 

Neurons in sensory systems convey information about physical stimuli in their spike trains. In vitro, single neurons respond precisely and reliably to the repeated injection of the same fluctuating current, producing regions of elevated firing rate, termed events. Analysis of these spike trains reveals that multiple distinct spike patterns can be identified as trial-to-trial correlations between spike times [1]. Finding events in data with realistic spiking statistics is challenging because events belonging to different spike patterns may overlap. We propose a method for finding spiking events that uses contextual information to disambiguate which pattern a trial belongs to. The procedure can be applied to spike trains of the same neuron across multiple trials to detect and separate responses obtained during different brain states. The procedure can also be applied to spike trains from multiple simultaneously recorded neurons in order to identify volleys of near synchronous activity or to distinguish between excitatory and inhibitory neurons. The procedure was tested using artificial data as well as recordings *in vitro* in response to fluctuating current waveforms.
